# Selective amplification of glucocorticoid anti-inflammatory activity through synergistic multi-target action of a combination drug

**DOI:** 10.1186/ar2602

**Published:** 2009-01-26

**Authors:** Grant R Zimmermann, William Avery, Alyce L Finelli, Melissa Farwell, Christopher C Fraser, Alexis A Borisy

**Affiliations:** 1 CombinatoRx, Incorporated, First Street, Cambridge, MA 02142, USA

## Abstract

**Introduction:**

Glucocorticoids are a mainstay of anti-inflammatory therapy, but significant adverse effects ultimately limit their utility. Previous efforts to design glucocorticoid structures with an increased therapeutic window have focused on dissociating anti-inflammatory transcriptional repression from adverse effects primarily driven by transcriptional activation. An alternative to this medicinal chemistry approach is a systems biology based strategy that seeks to amplify selectively the anti-inflammatory activity of very low dose glucocorticoid in immune cells without modulating alternative cellular networks that mediate glucocorticoid toxicity.

**Methods:**

The combination of prednisolone and the antithrombotic drug dipyridamole was profiled using *in vitro *and *in vivo *models of anti-inflammatory activity and glucocorticoid-induced adverse effects to demonstrate a dissociated activity profile.

**Results:**

The combination synergistically suppresses release of proinflammatory mediators, including tumour necrosis factor-α, IL-6, chemokine (C-C motif) ligand 5 (RANTES), matrix metalloproteinase-9, and others, from human peripheral blood mononuclear cells and mouse macrophages. In rat models of acute lipopolysaccharide-induced endotoxemia and delayed-type hypersensitivity, and in chronic models of collagen-induced and adjuvant-induced arthritis, the combination produced anti-inflammatory activity that required only a subtherapeutic dose of prednisolone. The immune-specific amplification of prednisolone anti-inflammatory activity by dipyridamole did not extend to glucocorticoid-mediated adverse effects, including corticosterone suppression or increased expression of tyrosine aminotransferase, *in vivo *after repeat dosing in rats. After 8 weeks of oral dosing in mice, treatment with the combination did not alter prednisolone-induced reduction in osteocalcin and mid-femur bone density, which are markers of steroid-induced osteoporosis. Additionally, amplification was not observed in the cellular network of corticotroph AtT-20/D16v-F2 cells *in vitro*, as measured by pro-opiomelanocortin expression and adrenocorticotropic hormone secretion.

**Conclusions:**

These data suggest that the multi-target mechanism of low-dose prednisolone and dipyridamole creates a dissociated activity profile with an increased therapeutic window through cellular network selective amplification of glucocorticoid-mediated anti-inflammatory signaling.

## Introduction

The robust anti-inflammatory effects of glucocorticoids are applied broadly in the clinical setting to treat diverse conditions, including rheumatic diseases, allergy, skin disorders, pulmonary conditions, cancer, transplant rejection, and even spinal cord injury. Unfortunately, the long-term clinical utility of glucocorticoids is limited by undesirable adverse effects, including suppression of the hypothalamus-pituitary-adrenal (HPA) axis, increased serum glucose, induction of osteoporosis and glaucoma, altered electrolyte balance, insomnia, and other behavioral alterations. Chronic treatment with even relatively low doses (for instance, 7.5 mg/day prednisolone) can lead to a subset of glucocorticoid-induced adverse effects [1,2]. Development of glucocorticoids with an improved therapeutic window has therefore been an area of focus for multiple groups.

The diverse effects of glucocorticoids are mediated by the glucocorticoid receptor (GR). Unliganded GR is retained in the cytosol by heat shock proteins that are released upon binding and activation by glucocorticoid [3]. Once activated the GR translocates to the nucleus and can bind directly to glucocorticoid response elements (GREs) as a homodimer, resulting in both activation and repression of transcription, depending on promoter structure and interaction with various co-activators and co-repressors. Additionally, activated GR can affect transcription through mechanisms independent of DNA binding that modulate the activity of other transcription factors, including nuclear factor-κB, activator protein-1, and STAT (signal transducer and activator of transcription) [4]. Finally, activated GR elicits a variety of transcription-independent effects through modulation of mRNA stability via 3'-untranslated region binding [5]. It is the integration of these diverse mechanisms, which affect multiple molecular targets, cells, and tissues, that results in the desirable anti-inflammatory activity and undesirable adverse effects of glucocorticoid treatment.

Efforts to dissociate the anti-inflammatory activity from the adverse effects of glucocorticoids have focused primarily on separating the DNA-binding-dependent (transcriptional activating) and DNA-binding-independent (transcriptional repressing) activities of activated GR. Dimerization-defective mutants of GR that lack DNA-binding activity can repress activator protein-1 mediated transcription, but they cannot activate transcription of GRE-regulated genes [6]. Glucocorticoid treatment can suppress local and systemic inflammation in homozygotic mice that express this GR^dim ^mutation, underscoring the importance of DNA-binding-independent mechanisms to the anti-inflammatory effect observed *in vivo *[7]. In contrast, many adverse effects of glucocorticoid treatment are due to DNA-binding-dependent activation (hyperglycemia, hypertension) or repression (suppression of HPA axis, osteoporosis) of transcription through activated GR homodimer binding to GREs [8,9]. A number of selective GR modulators or selective GR agonists (SEGRAs) have been developed that can dissociate anti-inflammatory activity from some of the classical glucocorticoid adverse effects [10-15].

Early attempts at steroid dissociation using medicinal chemistry have yielded mixed degrees of success because the anti-inflammatory activity and adverse effects of glucocorticoids do not break cleanly along the mechanistic lines of transcriptional repression and transcriptional activation. For example, adverse glucocorticoid effects including suppression of HPA axis, osteoporosis, and skin atrophy are probably induced, at least in part, by DNA-binding-independent repressive effects [8]. Similarly, the anti-inflammatory targets annexin-1 (lipocortin-1) [16], glucocorticoid-induced leucine zipper [17,18], and tristetraprolin [19] are positively regulated by DNA-binding-dependent transcriptional activating effects of glucocorticoids. Finally, macrophages from GR^dim ^mice exhibit a decreased potency of glucocorticoid suppression of IL-1β, monocyte chemotactic protein-1 (CCL2), macrophage inflammatory protein-2 (CXCL2), and interferon-gamma-inducible protein-10 (CXCL10) [20]. It is likely that effective dissociation of glucocorticoid action to enhance therapeutic index will require a careful tuning of both DNA-binding-dependent (transcriptional activating) and DNA-binding-independent (transcriptional repressing) effects to achieve an improved balance of desirable anti-inflammatory activity over induction of adverse effects [21]. This type of multi-parametric optimization presents a significant challenge to the medicinal chemistry approach to glucocorticoid dissociation.

An alternative approach to dissociation makes use of synergistic multi-target biology to amplify selectively the anti-inflammatory activity of glucocorticoids in immune cells without affecting glucocorticoid-induced adverse effects in alternative cellular networks [22,23]. The combined molecular effects of the antithrombotic agent dipyridamole and a very low dose of the glucocorticoid prednisolone create such an activity profile. Dipyridamole inhibits the activity of equilibrative nucleoside transporters and phosphodiesterases to increase cAMP and cGMP that block platelet activation, and it is used therapeutically in combination with low-dose aspirin for secondary stroke prevention [24]. Dipyridamole has also demonstrated anti-inflammatory activity using cell-based *in vitro *models [25]. The synergistic combination of prednisolone and dipyridamole suppresses tumor necrosis factor (TNF)-α secretion by lipopolysaccharide (LPS)-stimulated human peripheral blood mononuclear cells, as well as secretion of a unique set of cytokines, chemokines, and proteases by mouse bone-derived macrophages [26] (Fraser CC, unpublished data). In addition to suppressing the rheumatoid arthritis (RA)-modifying target TNF-α, the combination inhibits additional targets, including chemokine (C-C motif) ligand 5 (RANTES) and matrix metalloproteinase-9 (gelatinase-B), which are upregulated in RA synovium [27-30], and IL-6, which has been validated as a new target for the treatment of RA [31]. In RA, low-dose prednisolone treatment is generally considered to be a daily dose of 7.5 mg [32,33]. The combination of very low dose prednisolone (3 mg/day) and dipyridamole (400 mg/day) has exhibited statistically significant effects in human clinical trials of hand osteoarthritis [34] and RA (Kirwan JR, unpublished data). The selectivity of this synergistic combination was demonstrated by first measuring the activity of the combination in both acute and chronic models of inflammation in rats. The combination was then tested in various *in vivo *models of glucocorticoid-induced adverse effects, including suppression of the HPA axis marker corticosterone, induction of the gluconeogenesis gene tyrosine aminotransferase (TAT), and effects on markers of bone homeostasis. These data support a selective activity profile for this combination, in which dipyridamole amplifies the desired anti-inflammatory activity of prednisolone in immune cells without enhancing glucocorticoid action in alternative cellular networks that mediate adverse effects to generate an improved therapeutic index.

## Materials and methods

### Human peripheral blood mononuclear cell cytokine suppression assay

Compounds were obtained from Sigma-Aldrich (St. Louis, MO, USA), and stock solutions of appropriate concentration (in dimethyl sulfoxide) were serially diluted on master plates using liquid-handling automation and transferred to assay plates. Human buffy coat was obtained fresh daily from healthy donors and diluted in media supplemented with 10% fetal bovine serum (FBS; HyClone (Logan, UT, USA)) prior to stimulation with LPS (catalog number L-4130; Sigma-Aldrich) at 2 μg/ml and addition to assay plates. Plates were incubated for 18 hours at 37°C and 5% carbon dioxide. Supernatants were transferred to an ELISA plate coated with anti-TNF-α antibody (catalog number 551220; BD Pharmingen (San Diego, CA, USA)). Plates were then washed before probing with a second antibody (catalog number 554511; BD Pharmingen) and europium-labeled detection reagent (catalog number 1244-360; PerkinElmer (Waltham, MA, USA)). Raw data values of time-resolved fluorescence were converted to relative fractional inhibition (I = 1 – T/U) by comparing compound or combination treated values (T) with the median vehicle-alone level (U). Synergy is determined by comparing the combination's response to the Loewe additivity standard [35], and comparisons were made numerically using the combination index (CI) [36]. For example, CI_70 _= (C_X_/IC_70X_) + (C_Y_/IC_70Y_), where (C_X_/IC_70X_) for a mixture is the ratio of compound X concentration in a 70% effective mixture (C_X_) over its 70% inhibitory concentration when applied alone (IC_70X_).

### Rat endotoxemia model

Lewis (LEW/SsNHsd) rats (n = 8/group) were administered the appropriate test or control agent via oral gavage. Two hours after test or control substance administration (time = 120 minutes), animals were injected intraperitoneally with *Escherichia coli *serotype 0111:B4 LPS (Sigma-Aldrich). Control animals received a saline injection. Animals were euthanized by carbon dioxide asphyxiation 90 minutes after LPS administration. Serum samples were assayed for TNF-α levels using an ELISA (BioSource, Camarillo, CA, USA). All study procedures were approved by the CombinatoRx, Inc. Institutional Animal Care and Use Committee.

### Mouse delayed-type hypersensitivity model

CD-1 mice (n = 5/group) were sensitized with 2,4-dinitrofluorobenzene (DNFB) solution by application to the abdomen. Five days after application of DNFB, mice were administered test agents by oral gavage at the indicated doses (mg/kg). Two hours after the administration of the test agents, animals were challenged by painting the outer and inner surface of the left ear with DNFB. The right ear was painted with diluent (4:1 acetone/olive oil) as a control. Twenty-four hours after challenge, mice were anesthetized and the thickness of the DNFB-treated ear and the control ear were measured using electronic precision calipers to determine the change in thickness (mm).

### Rat collagen-induced arthritis model

Lewis (LEW/SsNHsd) rats (n = 12/group) were immunized with type-II collagen from newborn calf joints (Elastin Products Company, Inc., Owensville, MO, USA) emulsified in incomplete Freund's adjuvant (product number F5506; Sigma-Aldrich). Approximately 2 mg/kg collagen was given to all animals via intradermal injection on day 1 of the study. Two injections of 100 μl of collagen/adjuvant were made, one into the base of the tail and the other further up the back, separated by approximately 1.5 cm. A boost injection of the same material was given intradermally on day 6 of the study. Vehicle and test agents were administered via oral gavage. Dosing volume was 5 ml/kg and was adjusted weekly based on body weight measurements. Treatment period was from day 10 through day 27. Tibiotarsal joint thickness was measured using an electronic caliper on days 3, 6, 8, 13, 15, 17, 20, 22, 24 and 27. Change in joint thickness was calculated relative to the day 3 measurement. All study procedures were approved by the MDS Pharma Services (Bothell, WA, USA) Institutional Animal Care and Use Committee.

Louvain rats (n = 12/group) were immunized with solubilized type II collagen (1 mg/ml) in incomplete Freund's adjuvant injected intradermally into 15 sites on the back. Collagen-induced arthritis (CIA) developed over the next 10 days and test agents were administered every day by intragastric gavage from days 10 to 28 at the doses indicated (mg/kg). Arthritis severity was recorded daily for each hind paw using an integer scale from 0 to 4 to quantify the level of erythema and swelling (0 = normal; 4 = maximum). The sum of both hind paws (maximum score of 8) represented the severity of arthritis. Hind limbs were harvested at sacrifice (day 28) and scored by radiographic joint index on a scale from 0 to 3, based on soft tissue swelling, joint space narrowing, periosteal new bone formation, and presence of erosions and/or ankylosis (0 = normal; 3 = maximum joint destruction). The radiographic joint index represented the sum of both hind paws with a maximum score of 6. The experimental protocol conformed to the approved protocols of the UCLA Animal Care and Use Committee.

### Rat repeat dosing model

Lewis (LEW/SsNHsd) rats (n = 5/group) were weighed and placed into one of the six study groups. Body weights were recorded every other day throughout the study. Animals were dosed daily via oral gavage with test agents at volumes based on body weight progression throughout the study. On day 10, 2 hours after oral dosing, animals were euthanized via carbon dioxide asphyxiation. All study procedures were approved by the CombinatoRx, Inc. Institutional Animal Care and Use Committee.

Blood was collected and separated for corticosterone determination from serum (Diagnostic Systems Laboratories, Inc., Webster, TX, USA). Thymus and spleens were collected and weighed. Liver samples were removed and stored in RNAlater (Ambion, Austin, TX, USA) at 4°C. Liver samples were homogenized using TissueRuptor (Qiagen) and total RNA was isolated using the RNeasy-Plus Mini kit (Qiagen, Valencia, CA, USA). Equal amounts of total RNA were used for one step RT-PCR (QuantiTect Probe; Qiagen). Commercially available assay reagents (Taqman Gene Expression Assays; Applied Biosystems, Foster City, CA, USA) were used for detection of TAT and β-actin (endogenous control) mRNA, using the Applied Biosystems 7300 Real-Time PCR System.

### Mouse osteoporosis model

Mice (C57Bl/6) were randomized (n = 10/group) to treatment groups based on body weight before the start of dosing. Test agents were administered via oral gavage, with the exception of dexamethasone, which was administered via subcutaneous injection. All agents were dosed twice daily and new formulations prepared weekly for a total treatment period of 8 weeks. Animals were given two doses of calcein 10 mg/kg intraperitoneally 6 and 2 days before necropsy for fluorochrome labeling. Animals were anesthetized with isoflurane before necropsy, a terminal blood sample was collected, and serum was separated and stored frozen until analysis for bone markers. Femurs and lumbar vertebrae were also collected for dual energy X-ray absorptiometry, peripheral quantitative computed tomography and histomorphometry. All study procedures were approved by the MDS Pharma Services Institutional Animal Care and Use Committee.

### Corticotroph cAMP assay

AtT-20 cells were seeded at a density of 60,000 cells per well in a 96-well plate for determination of changes in cAMP levels in response to various treatment conditions. Cells were allowed to recover for 18 hours and then treated with dipyridamole (10 μmol/l), rolipram (10 μmol/l), or dimethyl sulfoxide control for 30 minutes at room temperature. Cells were then stimulated with corticotrophin-releasing factor (37.5 nmol/l), or control (vehicle), for 30 minutes at room temperature. cAMP levels were quantitated using the LANCE cAMP Detection Kit (PerkinElmer).

### Pro-opiomelanocortin expression and adrenocorticotropic hormone secretion assays

The murine anterior pituitary corticotroph cell line AtT-20/D16v-F2 was obtained from the American Type Culture Collection (Manassas, VA, USA) and maintained in Dulbecco's minimal essential medium (American Type Culture Collection) with 10% FBS, at 37°C with 5% carbon dioxide. To determine relative pro-opiomelanocortin (POMC) expression in AtT20 cells treated with prednisolone and/or dipyridamole, real-time RT-PCR was performed on cell lysates using the FastLane Cell RT-PCR kit (Qiagen). Commercially available assay reagents (Taqman Gene Expression Assays; Applied Biosystems) were used for detection of POMC β-actin (endogenous control) mRNA. Real time RT-PCR was done using the Applied Biosystems 7300 Real-Time PCR System. For ACTH secretion experiments, AtT20 cells were seeded in 24-well plates at a density of 125,000 cells/well in Dulbecco's minimal essential medium supplemented with 10% charcoal/dextran FBS (HyClone), and treated with prednisolone and/or dipyridamole. After 24 hours, the medium was refreshed with the same compound treatment in the absence or presence of corticotropin-releasing factor (CRF; 100 nmol/l). Three hours later (27 hours), culture medium was collected for evaluation of ACTH by ELISA (MD Biosciences, St. Paul, MN, USA).

## Results

### *In vitro *anti-inflammatory assays

**Figure 1:**
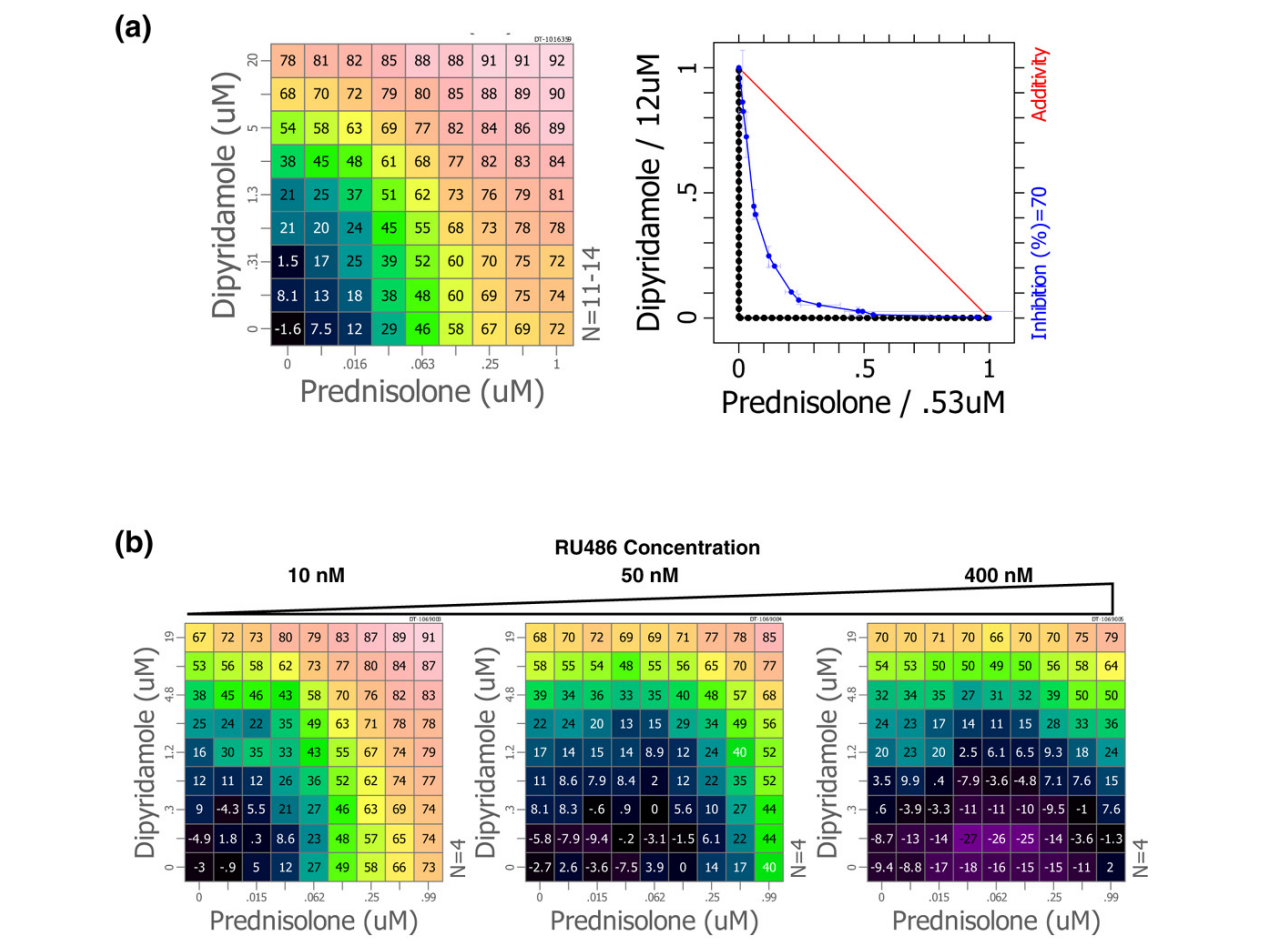
Synergistic anti-inflammatory activity of prednisolone and dipyridamole *in vitro*. **(a) **Dipyridamole and prednisolone were diluted orthogonally using a twofold serial dilution, and then combined to produce a drug combination dose-response matrix. The dose responses for prednisolone and dipyridamole as individual agents are located in the bottom row and left column, respectively. Combination doses fill out the matrix and component concentrations can be read from the row and column labels. The combination dose-response matrix was applied to lipopolysaccharide (LPS)-stimulated human peripheral blood mononuclear cells (PBMCs), and tumor necrosis factor (TNF)-α in the supernatant was measured by ELISA after 18 hours. Percentage inhibition of TNF-α secretion relative to vehicle-treated controls is indicated in each cell of the matrix and represented by a color scale, where warm colors indicate more inhibition (left). Isobolographic analysis of the inhibition matrix (blue line) compares the activity of the combination with a theoretical additive interaction (red line) at the 70% inhibition level (right). Synergistic interactions fall below the additivity threshold and approach the origin, and an antagonistic interaction would lie above the red additivity line. **(b) **Combination matrices were measured including a fixed dose of RU486 at the indicated concentration at each point in the corresponding dose response matrix. Percentage inhibition of LPS-induced TNF-α secretion relative to vehicle-treated controls is indicated in each cell of the matrix.

The combination of prednisolone and dipyridamole synergistically suppresses production of proinflammatory markers *in vitro*. The combination was discovered based on the observation of synergistic suppression of TNF-α from phorbol myristate acetate and calcium ionophore stimulated human peripheral blood mononuclear cells (PBMCs [see Figure S1 in Additional data file 1]). In a secondary assay the combination was found to synergistically suppress TNF-α secretion from LPS-stimulated PBMCs with a CI of 0.31 ± 0.02 (Figure 1a, left panel). Combinations with CI about 1 interact additively, such as would be expected when combining a drug with itself, and CI values below 1 indicate a synergistic interaction between the components [36]. Isobolographic analysis indicates that the synergistic effect of the combination allows reduction of the drug concentrations required to achieve 70% inhibition of TNF-α secretion by ten-fold for prednisolone and five-fold for dipyridamole (Figure 1a, right panel).

The activity of prednisolone and the combination effect is abolished by treatment with the GR antagonist RU486 at a concentration of 50 nmol/l, but dipyridamole activity is unaffected (Figure 1b). Antagonism by such a low dose of RU486 suggests that the effect may be mediated primarily by the GRE-dependent transcriptional-activating activity of dimerized GR.

### *In vivo *anti-inflammatory assays

**Figure 2:**
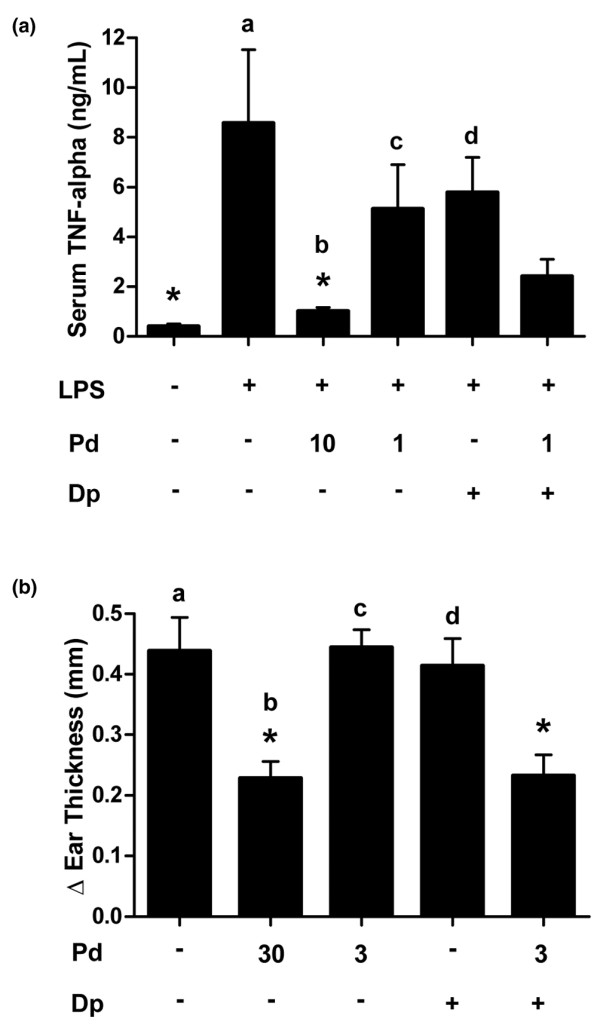
Prednisolone and dipyridamole combine to suppress acute inflammation *in vivo*. **(a) **Lewis rats were treated orally with compounds as indicated (mg/kg) for 2 hours before challenge with lipopolysaccharide (LPS). Serum was collected 90 minutes later and tumor necrosis factor (TNF)-α quantitated by ELISA. *P < 0.01 versus the LPS control; ^a^*P *= 0.06, ^b^*P *= 0.98, ^c^*P *= 0.81, ^d^*P *= 0.59 versus the combination. **(b) **Mice were sensitized with the chemical irritant 2,4-dinitrofluorobenzene (DNFB). Five days later animals were administered test agents by oral gavage at the indicated doses (mg/kg) and challenged on the ear with DNFB solution. Change in ear thickness was measured 24 hours after challenge (Δ ear thickness [mm]). *P < 0.05 versus the vehicle control; ^a^*P *= 0.02, ^b^*P *= 1.0, ^c^*P *= 0.02, ^d^*P *= 0.05 versus the combination. Dipyridamole was dosed at 150 mg/kg. Error bars are + standard error of the mean. Statistical comparison is by analysis of variance with Tukey. Dp, dipyridamole; Pd, prednisolone.

The combination of prednisolone and dipyridamole suppresses TNF-α in models of acute inflammation, and disease activity in CIA in rats. High-dose prednisolone (10 mg/kg) administered orally 2 hours before LPS challenge was able to significantly reduce serum TNF-α in an acute model of rat endotoxemia [37]. Prednisolone at 1 mg/kg, and dipyridamole at 150 mg/kg, yielded nonsignificant reductions in TNF-α release compared to the LPS control. The two agents combined yielded reduction in TNF-α release that was intermediate between the effects of low-dose and high-dose prednisolone (Figure 2a). These trends were also observed in repeats of the LPS challenge model.

High-dose prednisolone dosed orally at 30 mg/kg was able to suppress chemical hypersensitivity induced ear swelling (Figure 2b). A tenfold lower dose of prednisolone (3 mg/kg) and dipyridamole (150 mg/kg) as individual agents had no effect relative to vehicle-treated controls. The combination demonstrated efficacy equal to high-dose prednisolone, suggesting ten-fold amplification by dipyridamole of the anti-inflammatory activity of low-dose prednisolone in this acute model.

**Figure 3:**
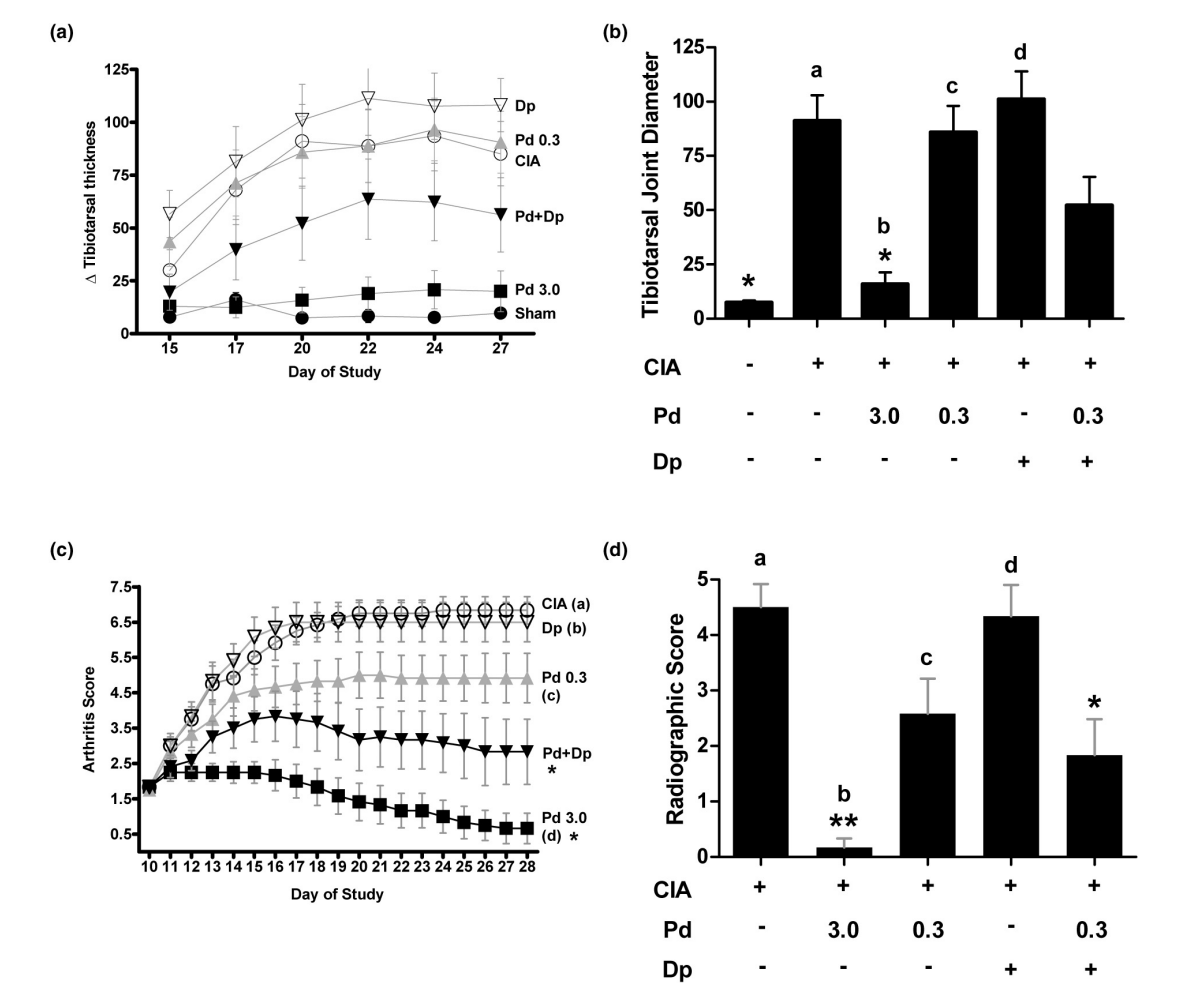
Prednisolone and dipyridamole combine to suppress collagen-induced arthritis *in vivo*. Collagen-induced arthritis (CIA) was developed in Lewis rats for 10 days before oral daily dosing with compounds as indicated (mg/kg) for the next 17 days. Change in hind limb tibiotarsal joint diameter relative to the day 3 measurement is reported **(a) **over the course of the study and **(b) **at study completion. *P < 0.001 versus the CIA control; ^a^*P *= 0.29, ^b^*P *= 0.10, ^c^*P *= 0.14, ^d^*P *= 0.004 versus the combination. **(c) **CIA was induced in Louvain rats for 10 days and test agents were administered orally once daily from days 10 to 28, as indicated (mg/kg). Arthritis severity was scored daily based on erythema and swelling. *P < 0.001 versus the CIA control; ^a^*P *= 0.0003, ^b^*P *= 0.001, ^c^*P *= 0.15, ^d^*P *= 0.12 versus the combination at day 28. **(d) **Hind limbs were scored by radiographic joint index after the completion of the study. **P < 0.0001, *P < 0.01 versus the CIA control; ^a^*P *= 0.005, ^b^*P *= 0.17, ^c^*P *= 0.84, ^d^*P *= 0.01 versus the combination at day 28. Dipyridamole was dosed at 150 mg/kg. Error bars are ± standard error of the mean, and statistical comparison is by analysis of variance with Tukey. Dp, dipyridamole; Pd, prednisolone.

Disease activity was also suppressed by the combination of prednisolone and dipyridamole in CIA models in Lewis and Louvain rats. To test the activity of the combination in this model of chronic inflammation, rats were sensitized with collagen and hind paw arthritis developed over the next 10 days [38,39]. Lewis rats treated with prednisolone (3 mg/kg orally) exhibited negligible tibiotarsal joint swelling compared with animals that were not subjected to collagen induction, and rats treated with 0.3 mg/kg prednisolone or 150 mg/kg dipyridamole showed similar levels of tibiotarsal inflammation to CIA-induced, vehicle-treated controls (Figure 3a,b). Tibiotarsal joint swelling trends were maintained throughout the study. The combination of prednisolone and dipyridamole (0.3/150 mg/kg) yielded a significant reduction in swelling compared with dipyridamole alone, and tibiotarsal swelling in the combination group was intermediate between the low-dose and high-dose prednisolone groups at all points in the study.

The CIA model was also repeated in Louvain rats [39], and arthritis score was measured daily from days 10 to 28 (Figure 3c,d). At the conclusion of the study (day 28) animals treated with vehicle or dipyridamole had arthritis scores of about 6.5, which were significantly different from the scores in the combination and high-dose prednisolone groups. Combination treated animals (0.3/150 mg/kg) had an average arthritis score of 2.8, which was intermediate between the effect of low-dose prednisolone (4.9) and high-dose prednisolone (0.7), suggesting that dipyridamole can amplify the activity of low-dose prednisolone in suppressing erythema and joint swelling. Radiographic analysis of the hind limbs at the conclusion of the study indicated that the combination significantly reduced tissue damage relative to the vehicle control, and was similar to low-dose steroid alone on measures of joint space narrowing and the presence of erosions and/or ankylosis (Figure 3d).

An additional test of the combination was conducted in an adjuvant-induced arthritis model [40], and similar anti-inflammatory activity of the combination was observed. A higher dose of dipyridamole (300 mg/kg) was required to observe the effect in this particular model. Tissue was collected and prepared for histologic evaluation of tarsal and phalangeal joints based on inflammatory infiltrate, pannus formation, and cartilage and bone degeneration. The combination of prednisolone and dipyridamole achieved reduction in cartilage damage in the tibiotarsal joint similar to that observed for the high-dose prednisolone (5 mg/kg) positive control group. The combination strongly suppressed cartilage damage in the phalangeal joints as well as inflammation, pannus formation, and bone damage, as indicated by histologic analysis [see Figure S2 in Additional data file 1].

### *In vivo *safety assays

**Figure 4:**
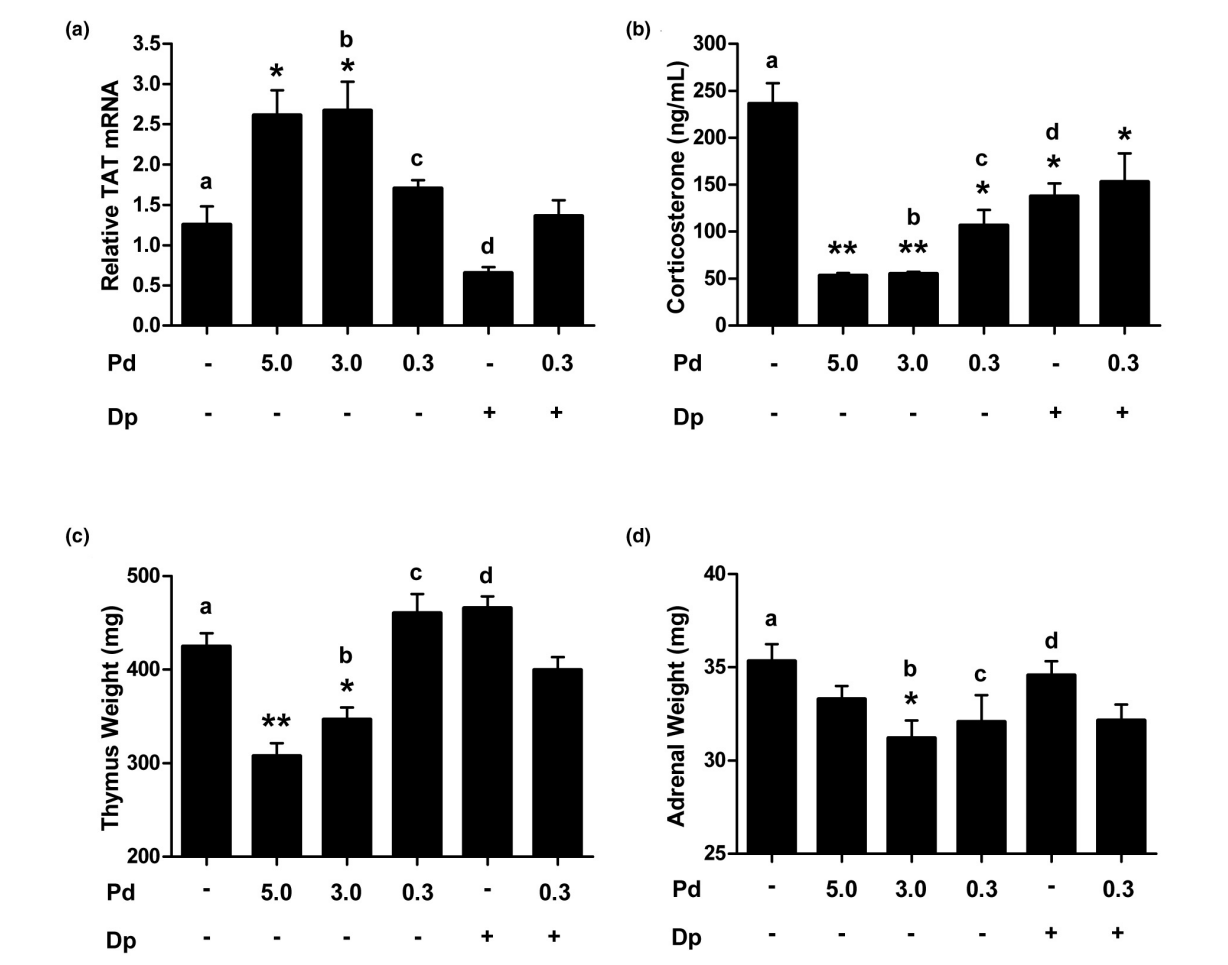
Dipyridamole does not alter the low-dose prednisolone safety profile. **(a) **Tyrosine aminotransferase (TAT) mRNA levels from liver were evaluated by RT-PCR after 10 days of repeat dosing in Lewis rats as indicated. β-Actin was used as the endogenous control, and results are shown as fold increase in TAT mRNA over vehicle. *P < 0.01 versus the vehicle control, ^a^*P *= 1.0, ^b^*P *= 0.01, ^c^*P *= 0.90, ^d^*P *= 0.30 versus the combination. **(b) **Corticosterone levels in serum were evaluated by ELISA after 10 days of chronic dosing. **P < 0.0001, *P < 0.05 versus the vehicle control; ^a^*P *= 0.03, ^b^*P *= 0.01, ^c^*P *= 0.44, ^d^*P *= 0.98 versus the combination. **(c) **Thymus weight was measured at the conclusion of the study. **P < 0.0001, *P < 0.01 versus the vehicle control; ^a^*P *= 0.83, ^b^*P *= 0.14, ^c^*P *= 0.06, ^d^*P *= 0.03 versus the combination. **(d) **Adrenal weights were also evaluated upon study completion. *P < 0.05 versus the vehicle control; ^a^*P *= 0.21, ^b^*P *= 0.98, ^c^*P *= 1.0, ^d^*P *= 0.50 versus the combination. Dipyridamole was dosed at 300 mg/kg. Error bars are + standard error of the mean, and statistical comparison is by analysis of variance with Tukey. Dp, dipyridamole; Pd, prednisolone.

The observed amplification by dipyridamole of prednisolone anti-inflammatory activity did not extend to classical glucocorticoid adverse effects. Lewis rats were treated once daily for 10 days with oral dose groups of prednisolone identical to those used in the CIA model. The amplifying dose of dipyridamole was increased two-fold to 300 mg/kg for the safety studies. This increased dose of dipyridamole had demonstrated increased anti-inflammatory effect in some models. At the conclusion of dosing, appropriate tissues were harvested to measure safety parameters. Liver mRNA levels of TAT, a marker of glucocorticoid-activated glucose metabolism, were evaluated by RT-PCR after repeated treatment with prednisolone and dipyridamole alone, and in combination. Animals treated daily with 3 or 5 mg/kg of prednisolone for 10 days experienced a 2.6-fold increase in the expression of TAT mRNA in the liver (Figure 4a). In contrast, animals treated with 0.3 mg/kg prednisolone exhibited a 1.7-fold increase in TAT mRNA with oral daily dosing. Treatment with the combination of prednisolone and dipyridamole (0.3/300 mg/kg) resulted in significantly lower TAT mRNA levels than in the high-dose prednisolone (3 mg/kg) treatment group, but was no different from the effect of the component prednisolone dose (0.3 mg/kg) alone (Figure 4a).

Glucocorticoids can suppress products of the HPA axis including, serum corticosterone. After chronic treatment of Lewis rats with the combination or individual components for 10 days, serum was collected to quantitate levels of corticosterone. The 3 and 5 mg/kg prednisolone groups exhibited significantly suppressed serum corticosterone (Figure 4b). Prednisolone at 0.3 mg/kg had less of an effect on corticosterone, and this effect was not amplified when applied in combination with dipyridamole (300 mg/kg). Thymus and adrenal gland weights were also measured following chronic dosing. Prednisolone at 3 and 5 mg/kg suppressed thymus weight, but 0.3 mg/kg prednisolone alone or in combination had no significant effect on thymus weight compared with vehicle control (Figure 4c). The effect of the combination (0.3/300 mg/kg) on adrenal weight was identical to the effect of 0.3 mg/kg prednisolone alone, and neither was significantly different from the vehicle control group (Figure 4d).

**Figure 5:**
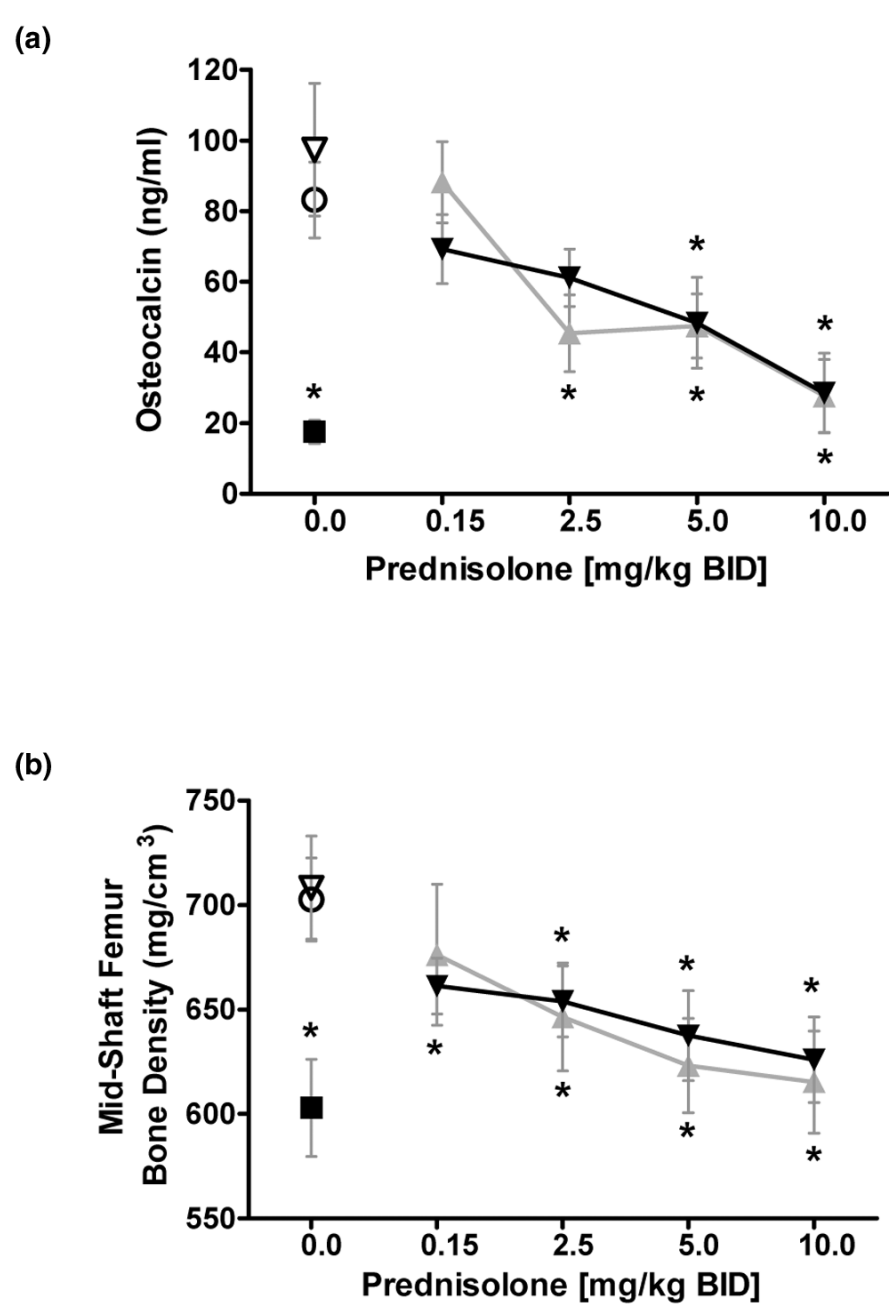
Dipyridamole does not amplify prednisolone effects on surrogate markers of osteoporosis. BL/6 mice were dosed twice daily with test agents for a total of 8 weeks to measure effects on markers of bone homeostasis. **(a) **Serum was collected at the end of the study and osteocalcin was measured by ELISA. **(b) **End of study mid-shaft femur bone density was measured by flurochrome labeling, sectioning, and peripheral quantitative computed tomography. Prednisolone alone (grey curve); prednisolone in combination with dipyridamole twice daily (black curve); dipyridamole alone and vehicle control are indicated with open triangle and open circle, respectively; sub-cutaneous dexamethasone (5 mg/kg once daily) positive control is indicated with a black square. *P < 0.05 versus the vehicle control. Dipyridamole was dosed at 37.5 mg/kg twice daily in this study (allometrically scaled from a rat total daily dose of 150 mg/kg). Error bars are ± standard deviation, and statistical comparison is by analysis of variance with Tukey.

Chronic treatment with glucocorticoids can alter expression of various markers of osteoporosis, including osteocalcin and structural measures of bone density and quality. Female mice were treated orally twice daily with various doses of prednisolone alone, dipyridamole, or the combination of varying doses of prednisolone with dipyridamole, for 8 weeks before quantitation of these surrogate markers of osteoporosis. Dexamethasone (5 mg/kg once daily) significantly reduced osteocalcin and mid-shaft femur bone density compared with vehicle-treated controls. Prednisolone was associated with a dose-dependent reduction in osteocalcin and mid-femur bone density that was not altered by the addition of dipyridamole (37.5 mg/kg twice daily; Figure 5).

### *In vitro *corticotroph assays

**Figure 6:**
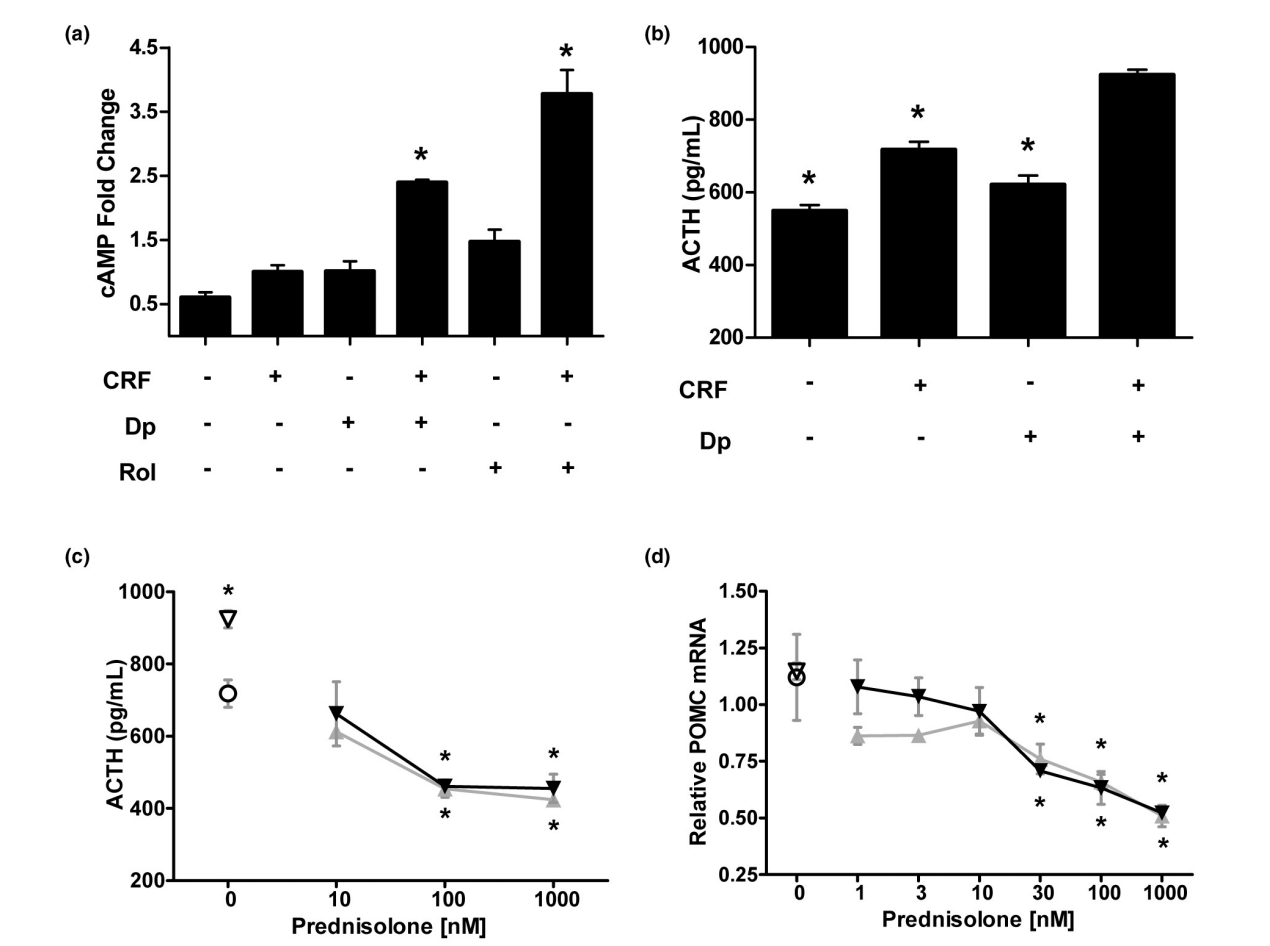
Dipyridamole does not amplify suppression of markers of the HPA axis *in vitro*. **(a) **AtT-20 corticotroph cells were pretreated with dipyridamole (Dp; 10 μmol/l) or rolipram (Rol; 10 μmol/l) as indicated. Cells were stimulated with corticotropin-releasing factor (CRF) or vehicle control before quantitating cAMP levels. Error bars are + standard deviation (SD). *P < 0.01 versus CRF alone. **(b) **AtT-20 cells were pretreated with Dp or control for 24 hours. Medium was refreshed with compounds, and then stimulated with CRF or vehicle control for an additional 3 hours. Medium was collected for determination of ACTH levels by ELISA. Error bars are + SD. *P < 0.001 versus CRF + Dp. **(c) **AtT-20 cells were pretreated with increasing doses of prednisolone in the absence (grey curve) or presence (black curve) of Dp (10 μmol/l) for 24 hours. Medium was refreshed with compounds plus CRF. After 3 hours, ACTH levels were determined by ELISA. *P < 0.001 versus vehicle alone. **(d) **AtT-20 cells were incubated with increasing doses of prednisolone in the absence (grey curve) or presence (black curve) of Dp for 24 hours. POMC mRNA levels were determined by RT-PCR analysis, using β-actin as the endogenous control. Dp alone (10 μmol/l) and vehicle control responses are indicated with an open triangle and circle, respectively. Error bars are ± SD, and statistical analysis is by analysis of variance with Tukey. HPA, hypothalamus-pituitary-adrenal.

The observed amplification by dipyridamole of the anti-inflammatory activity of prednisolone did not extend to suppression *in vitro *of the POMC gene and suppression of secreted ACTH from corticotroph cells. The effects of prednisolone, dipyridamole, and the combination were measured in the murine anterior pituitary cell line AtT-20/D16v-F2 (AtT-20), a well studied corticotroph model system [41]. The relative amount of cAMP was increased in the AtT-20 cells by 1.5-fold after treatment with dipyridamole and CRF stimulation (Figure 6a). The prototypic phosphodiesterase (PDE) 4 inhibitor rolipram increased cAMP by three-fold under these conditions. CRF stimulation increased ACTH secretion in untreated control cells after 3 hours, and pretreatment with dipyridamole (10 μmol/l) significantly increased ACTH release compared with CRF stimulation alone (Figure 6b). AtT-20 cells were pretreated with prednisolone, dipyridamole, or the combination for 24 hours, and then stimulated with CRF (100 nmol/l) to induce ACTH secretion. Pretreatment with prednisolone reduced ACTH secretion compared with the CRF-stimulated control, and the stimulatory effects of dipyridamole (10 μmol/l) on ACTH secretion were not observed in combination with any dose of prednisolone (Figure 6c). Prednisolone decreased POMC mRNA expression, with a maximum decrease of about 50% observed at the 24-hour time point (Figure 6d). The addition of dipyridamole (10 μmol/l) did not amplify the effect of prednisolone on POMC mRNA levels, and was able to compensate for the suppressive effect of very-low-dose prednisolone.

## Discussion

The potent anti-inflammatory activity and disease-modifying effects of glucocorticoids are well documented [42], but safety concerns observed with chronic dosing [2] have created a desire for safer glucocorticoids with an expanded therapeutic window. Many groups are pursuing this goal with a medicinal chemistry approach. Significant progress has been made by identifying novel GR ligands that retain substantial anti-inflammatory activity while reducing key glucocorticoid-induced adverse effects [10-15]. The success of the dissociated ligands developed to date is impressive, given the extreme complexity of the GR system and the challenge of developing low-molecular-weight compounds that retain desirable activities of the native ligand while selectively eliminating undesirable effects. Unfortunately, these ligands generally retain unacceptable activity on one or more adverse effect measures, suggesting that an alternative approach to glucocorticoid dissociation may be required.

A multi-component therapeutic can be envisaged that leverages systems biology to amplify glucocorticoid activity selectively in the network context of inflammatory cells over alternative cellular networks that mediate adverse effects. In this multi-target approach, an enhancing agent is used to sensitize the immune cell network to the effects of very-low-dose prednisolone by modulation of intersecting signaling pathways that selectively amplify the anti-inflammatory activity of the glucocorticoid. The divergent molecular context of cellular networks that mediate glucocorticoid adverse effects (for example, corticotrophs or hepatocytes) do not support amplification, and therefore the safety profile of the very-low-dose glucocorticoid is maintained. At the organism level, the effects of the combination produce the desired anti-inflammatory activity of a glucocorticoid with an enhanced therapeutic window.

The combination of prednisolone and the antithrombotic agent dipyridamole synergistically suppresses the secretion of TNF-α and other proinflammatory mediators from human PBMCs stimulated with LPS (Figure 1a), but also phorbol myristate acetate/Ionomycin-stimulated and α CD3/α CD28-stimulated cultures [see Figure S1 in Additional data file 1]. Because of the multi-target action of the components, the anti-inflammatory activity is achieved at doses where prednisolone and dipyridamole have marginal activity as individual agents. The components of the combination, including dipyridamole, are known to suppress TNF-α individually [43], but the synergistic combination effect was unexpected [26]. In secondary assays the combination was found to inhibit production of a unique profile of cytokines, chemokines, and proteases from LPS-stimulated mouse bone-derived macrophages, including synergistic suppression of TNF-α, IL-6, and chemokine (C-C motif) ligand 5, which are validated targets in rheumatic disease (Fraser CC, unpublished data).

The activity of prednisolone and the synergy with dipyridamole was blocked *in vitro *by the GR antagonist RU486 (Figure 1b), demonstrating that the anti-inflammatory effect of the combination is, at least in part, GR dependent and may require dimerization and transcriptional activation. GRE-independent repression of nuclear factor-κB mediated transcription by glucocorticoid is not significantly antagonized by 10–100 nmol/l RU486, but GRE-dependent transcription is strongly inhibited [44]. This result potentially differentiates the activity of the combination from the dissociated GR ligands that are more effective at transrepression than transactivation [12,13,15]. The combination of prednisolone and dipyridamole, but not the components alone, has been shown to upregulate expression of glucocorticoid-induced leucine zipper and dual-specificity phosphatase-1 (DUSP1/MKP1) mRNA in LPS-stimulated mouse macrophages (Fraser CC, unpublished data). DUSP1 induction has been shown in some systems to require GR dimerization [45], but this was not observed in GR^dim ^mouse macrophages [46]. Together, these data suggest that transcriptional activation may make an important contribution to the anti-inflammatory activity of the combination.

The combination of prednisolone and dipyridamole was found to have anti-inflammatory activity in both acute and chronic models of inflammation *in vivo*. Low-dose prednisolone and dipyridamole had minimal effect as single agents, but the combination was able to suppress serum TNF-α in an LPS-challenge model, and ear swelling in the delayed-type hypersensitivity model (Figure 2). These data suggest that the molecular effect of dipyridamole may amplify the anti-inflammatory activity of low-dose prednisolone between three- and ten-fold *in vivo*. The ability of low-dose prednisolone to suppress disease activity in chronic models of arthritis was also amplified by dipyridamole. The arthritis score reduction for the combination was 2.1 units (4.9 for low-dose prednisolone and 2.8 for the combination), or one half the reduction observed between low-dose (0.3 mg/kg) and high-dose (3 mg/kg) prednisolone (4.9 – 0.7 = 4.2). These results suggest that amplification may be in the range of five-fold for the arthritis models, assuming a linear dose response (Figure 3). Prednisolone and dipyridamole also combine to suppress histologic markers of inflammation in an adjuvant-induced model of arthritis. Interestingly, the effect of combination was most pronounced in the phalangeal joints, where clinically relevant suppression of histologic markers of inflammation, and bone and cartilage degeneration were observed (see Figure S2 in Additional data file 1). However, this model required a higher dose of dipyridamole (300 mg/kg) that also demonstrated significant activity. The tissue-sparing effect of the combination may stem from the potent suppression of proinflammatory mediators from macrophages (Fraser CC, unpublished data). The activity of the combination observed in these animal models suggests that the molecular effects of dipyridamole can amplify the subtherapeutic anti-inflammatory activity of very-low-dose prednisolone to produce a disease-modifying effect.

Dipyridamole was not found to amplify the effect of prednisolone on markers of glucocorticoid-induced toxicity under the stringent conditions of repeat daily dosing *in vivo*. Increased gluconeogenesis leading to hyperglycemia is a common side effect of chronic glucocorticoid treatment [2]. The gluconeogenic potential of prednisolone and dipyridamole was evaluated by measuring the effect on the classical transcriptional activation target TAT by RT-PCR analysis of liver tissue from rats treated with the combination or its components. The effect of low-dose prednisolone was not altered by simultaneous dosing with 300 mg/kg dipyridamole (Figure 4a), suggesting no enhancement of the effect of prednisolone in hepatocytes. This result suggests that positive GRE-mediated glucocorticoid adverse effects, including hyperglycemia, may not be amplified by simultaneous dipyridamole treatment. Adrenal insufficiency is another common adverse effect of chronic glucocorticoid treatment [8], caused by the complex negative feedback control of the system. Prednisolone dose-dependently reduced serum corticosterone, and dipyridamole did not further suppress the effect of low-dose prednisolone (Figure 4b). This result suggests that the anti-inflammatory synergy observed between prednisolone and dipyridamole does not extend to the undesirable suppression of the HPA axis. Thymus and adrenal weights were also monitored in this study, and high-dose steroid was found to reduce these organ weights after once-daily dosing for 10 days. No significant effects relative to the vehicle control were observed for the component doses alone or the combination.

Dipyridamole did not amplify prednisolone-induced effects on markers of bone degradation *in vivo*. Osteoporosis is a common adverse effect of chronic treatment with glucocorticoids, and loss of bone from spine and hip is estimated at 1.5% per year with a daily dose of 9 mg prednisolone or equivalent [2]. Dipyridamole demonstrated no effect as a single agent and did not alter the prednisolone dose response for osteocalcin or mid-shaft femur bone density, suggesting a lack of combination effect on these markers (Figure 5) in mice treated for 8 weeks. The dipyridamole dose of 37.5 mg/kg twice daily in this study was allometrically scaled from the total daily dose of 150 mg/kg used in the rat CIA studies, in which dipyridamole was found to amplify the anti-inflammatory activity of low-dose prednisolone. Osteocalcin is a marker of bone formation that is known to be suppressed by glucocorticoids, caused by a negative GRE in the promoter [47]. These data suggest that dipyridamole does not amplify DNA-binding-dependent (GRE-mediated) transrepressive effects of low-dose glucocorticoid treatment. The results from this mouse study indicate that the immune-selective action of dipyridamole may not amplify the osteoporotic effect and fracture risk associated with very-low-dose prednisolone [48] in the clinical setting with chronic dosing.

To further explore the HPA axis effect of the combination, the individual effects of dipyridamole were characterized in the unique signaling network of AtT20 corticotroph cells. Dipyridamole treatment has been shown to inhibit PDE activity (PDE-2, -4, -10, and -11) and increase cAMP levels [49,50]. Dipyridamole treatment of AtT-20 cells results in a modest 1.5-fold increase in cAMP relative to CRF stimulation alone, but was less effective than the threefold increase observed with the potent PDE-4 inhibitor rolipram (Figure 6a). Increased cAMP was previously shown to increase ACTH secretion in AtT20 cells [51], and dipyridamole treatment was found to induce a small but significant increase in CRF-dependent ACTH secretion (Figure 6b), which may be due to increased cAMP. Interestingly, dipyridamole does not increase cAMP in mouse bone-derived macrophages (Fraser CC, unpublished data), suggesting a differential effect of dipyridamole in the immune cell network context as compared with the corticotroph network of the AtT20 cells. Activated GR is also known to activate a negative GRE and to antagonize the effect of cAMP-induced transcription factors to suppress POMC expression [52]. We observed a prednisolone dose-dependent decrease in both POMC expression and ACTH secretion from the AtT20 cells *in vitro*, and this response was unaffected by the addition of dipyridamole (Figure 6c,d). These experiments demonstrate a lack of synergy between prednisolone and dipyridamole on POMC expression or ACTH secretion in corticotroph cells, unlike the synergistic anti-inflammatory activity of the combination observed in human PBMCs, mouse macrophages, and in rat arthritis models. The differential response to combination treatment is probably due to differences in cellular network context, including expression levels and/or spatial relationships of molecular targets, activation state of relevant signaling pathways, and cell type-specific differences in transcription factor expression or activation. Defining the set of targets responsible for mediating the selectivity of the combination for immune cell networks over alternative cell contexts is the focus of ongoing research.

The success of the SEGRAs and selective GR modulators developed to date is impressive, given the extreme complexity of the GR system. SEGRAs have been identified that potently suppress irritant dermatitis [10,11,13], carrageenan-induced and adjuvant-induced arthritis [12], and CIA [15], with improved tolerability relative to body weight loss and elevation of blood glucose. For example, the dissociated ligand ZK 216348 exhibited no increase in serum glucose in fasted rats 6 hours after dosing at doses up to 30 mg/kg, as compared with an approximately 60% increase for prednisolone at 10 mg/kg [13]. TAT activity in these rats was minimally increased by acute dosing of ZK 216348, compared with a six-fold increase by prednisolone at an equivalent dose of 10 mg/kg [13]. Similarly, the dissociated GR ligand LGD5552 was demonstrated to be about ten-fold less potent than prednisolone in suppressing bone formation rate in a 4-week study [15]. Unfortunately, the complexity of the GR signaling network that controls transcriptional repression and activation, and the post-transcriptional effects of activated GR make it extremely challenging to develop low-molecular-weight compounds that retain the desirable activities of the native ligand while selectively eliminating undesirable effects.

Dissociation of anti-inflammatory effects and adrenal insufficiency has been especially problematic for the dissociated GR ligand approach. Glucocorticoid release from the adrenal is regulated by both DNA-binding-dependent and -independent (transrepressive) effects of the GR [53]. It may be that all dissociated GR ligands with transrepressive effects equivalent to glucocorticoids will display some degree of undesirable effect on the HPA axis. For example, acute subcutaneous treatment of rats with ZK 216348 resulted in ACTH suppression with potency equivalent to that of prednisolone [13]. Indeed, glucocorticoid activity and safety studies in GR^dim ^mice suggest that not all anti-inflammatory actions of activated GR are retained in the absence of dimerization, and neither are all glucocorticoid-induced adverse effects eliminated [54]. Dipyridamole was not found to amplify corticosterone suppression by low-dose prednisolone in the rat repeat dosing study (Figure 4b), and did not alter the prednisolone dose response for suppression of POMC and ACTH in the corticotroph cell line (Figure 6c,d). These results suggest that cellular network selective amplification of glucocorticoid activity by the multi-target mechanism of low-dose prednisolone and dipyridamole may provide a solution to the challenging problem of dissociating glucocorticoid-induced HPA axis suppression.

## Conclusion

There is an increased awareness in the drug discovery industry of the need for novel therapeutics that address the systems biology of disease, and a nascent trend exists that is moving away from the one-drug, one-target paradigm toward a more pathway-focused or multi-target approach to drug discovery [22,55-57]. Glucocorticoid dissociation efforts to date have focused on modulating GR dimerization or interaction with co-activators and co-repressors to separate the desirable and undesirable effects of glucocorticoids. The systems biology approach presented here exploits multi-target action to amplify glucocorticoid activity selectively in the unique network context of inflammatory cells, rather than attempting to dissect various aspects of GR biology. This approach is advantageous because the anti-inflammatory activity of the combination is therefore derived from amplification of native glucocorticoid action, be it transrepressive or transactivating. Cellular networks that mediate traditional glucocorticoid adverse effects do not support amplification of the low-dose glucocorticoid effect due to the absence of nodes or differential signaling pathway interactions, resulting in the selective action and an increased therapeutic window. The multi-target action of combination drugs may provide a general approach to achieve cell-type specific therapeutic effects.

## Abbreviations

ACTH: adrenocorticotropic hormone; CCL2: monocyte chemotactic protein-1; CI: combination index; CIA: collagen-induced arthritis; CRF: corticotropin-releasing factor; CXCL2: macrophage inflammatory protein-2; CXCL10: interferon-gamma-inducible protein-10; DNFB: 2,4-dinitrofluorobenzene; DUSP1: dual-specificity phosphatase-1; ELISA: enzyme linked immunosorbent assay; FBS: fetal bovine serum; GR: glucocorticoid receptor; GRE: glucocorticoid response element; HPA: hypothalamus-pituitary-adrenal; IL: interleukin; LPS: lipopolysaccharide; PBMC: peripheral blood mononuclear cell; PDE: phosphodiesterase; POMC: pro-opiomelanocortin; RA: rheumatoid arthritis; RT-PCR: reverse transcription polymerase chain reaction; SEGRA: selective glucocorticoid receptor agonist; TAT: tyrosine aminotransferase; TNF: tumor necrosis factor.

## Competing interests

The authors are employed by CombinatoRx, Incorporated.

## Authors' contributions

GRZ, WA, ALF, MF, CCF, and AAB designed the research and experiments. WA, ALF, and MF performed the experiments. GRZ, WA, ALF, MF, CCF, and AAB analyzed the data. GRZ wrote the manuscript.

## Supplementary Material

Additional file 1 A Word file containing Figures S1 and S2. Figure S1 reports the dose-response matrix data for the inhibition of TNF-α release from human PBMCs stimulated with phorbol myristate acetate (PMA)/ionomycin by the combination of prednisolone and dipyridamole. Figure S2 shows the inhibition of histologic markers of inflammation by the combination in a rat adjuvant-induced arthritis model. Click here for file
