# Supplementary material for: Selective amplification of glucocorticoid anti-inflammatory activity through synergistic multi-target action of a combination drug
Source: Arthritis Res Ther. 2009 Jan 26;11(1):R12. doi: 10.1186/ar2602 (PMC2688244; doi:10.1186/ar2602)
Supplement: Additional file 1 — A Word file containing Figures S1 and S2. Figure S1 reports the dose-response matrix data for the inhibition of TNF-α release from human PBMCs stimulated with phorbol myristate acetate (PMA)/ionomycin by the combination of prednisolone and dipyridamole. Figure S2 shows the inhibition of histologic markers of inflammation by the combination in a rat adjuvant-induced arthritis model. [file ar2602-S1.doc]

**Supplementary Figures**

|  |  |
| --- | --- |

**Figure S1. Synergistic anti-Inflammatory activity of prednisolone and dipyridamole *in vitro***

Dipyridamole and prednisolone were diluted orthogonally using a two-fold serial dilution, and then combined to produce a drug combination dose-response matrix. The dose-responses for prednisolone and dipyridamole as individual agents are located in the bottom row and left column respectively. Combination doses fill out the matrix and component concentrations can be read from the row and column labels. The combination dose-response matrix was applied to phorbol myristate acetate and calcium ionophore (PMA/I) stimulated human PBMCs, and TNFα in the supernatant was measured by ELISA after 18 hours. Percent inhibition of TNFα secretion relative to vehicle-treated controls is indicated in each cell of the matrix, and represented by a color scale where warm colors indicate more inhibition (Left). Isobolographic analysis of the inhibition matrix (blue line) compares the activity of the combination to a theoretical additive interaction (red line) at the 50% inhibition level (Right). Synergistic interactions fall below the additivity threshold and approach the origin, and an antagonistic interaction would lie above the red additivity line.

**A**

**B**


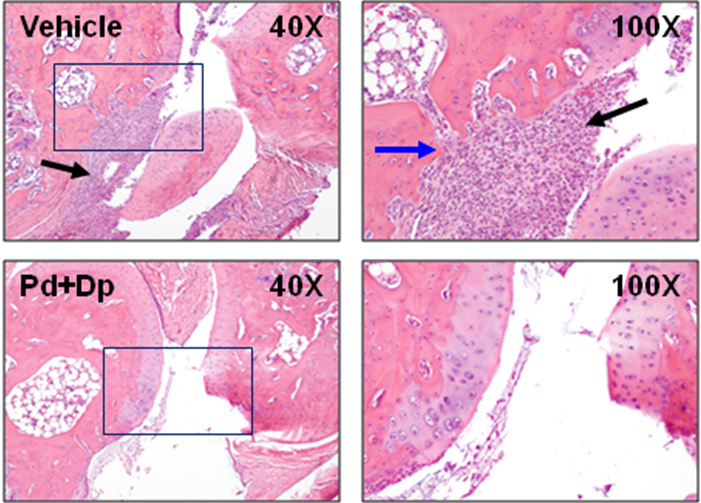


**Figure S2. Prednisolone and dipyridamole combine to suppress histological markers of inflammation.**

Lewis (LEW/SsNHsd) rats (N=16/group) were treated on day 1 with heat killed M. butyricum (Difco, Lot # 7171144) suspension in mineral oil injected intradermally into the base of the tail of each animal. Non-induced controls received an intradermal injection of mineral oil alone. Rats were randomized on day 11, and dosed once daily via oral gavage as indicated (mg/kg) on days 11-21. Tibiotarsal joints were measured 3 times per week using electronic calipers to measure disease activity (data not shown). After final joint assessment on day 22, animals were euthanized via carbon dioxide asphyxiation and the hind tibiotarsal joints were collected for histological analysis. Left hind limbs were cut at mid-metatarsal, decalcified, paraffin embedded, and 5 um sections were cut at 250 um increments and stained with hematoxylin and eosin. Histological analysis was conducted by a board-certified veterinary pathologist blinded to treatment groups. All study procedures were approved by the CombinatoRx, Inc. Institutional Animal Care and Use Committee. A) Histological evaluation of phalangeal cartilage damage at the conclusion of the study (day 22) is presented. a - P = 0.02, b - P = 0.04 versus the combination. Dipyridamole was dosed at 300 mg/kg. Error bars are +SEM, and statistical comparison is by two-tailed Mann-Whitney. B) Representative photomicrographs of the phalangeal joints from vehicle-treated or combination treated animals. Micrograph pairs are from the same section at low (40X) and high (100X) magnification. Black arrows indicate areas of inflammation and cartilage damage. The blue arrow indicates an area of bone damage. Phalangeal joint inflammation, cartilage, and bone damage were suppressed in animals treated with the combination of prednisolone and dipyridamole (Pd+Dp at 0.3/300 mg/kg) compared to vehicle-treated controls.
